# 1330. Pediatric infective endocarditis at a referral children’s hospital during 19-year period: Trends and Outcomes

**DOI:** 10.1093/ofid/ofac492.1160

**Published:** 2022-12-15

**Authors:** Ahmad Shawaqfeh, Shipra Gupta, Basim Asmar, Nahed M Abdel-Haq

**Affiliations:** Children's Hospital of Michigan, Detroit, Michigan; West Virginia University, Morgantown, West Virginia; Children's Hospital of Michigan. Central Michigan University, Detroit, Michigan; Children's Hospital of Michigan. Central Michigan University, Detroit, Michigan

## Abstract

**Background:**

We noted an increase in infective endocarditis (IE) cases in recent years. The purpose of the study was to examine the changes in incidence, risk factors, microbiology, complications and outcome of IE in our patient population.

**Methods:**

Records of children < 18 yrs with discharge diagnosis of IE during 2002-2020 at Children’s Hospital of Michigan, Detroit were reviewed. Modified Duke criteria were used to determine “definite” and “possible” IE cases.

**Results:**

101 patients with IE were identified, representing annual incidence of 4.9/10,000 admissions. During 2002-2011 (early period), the incidence was 2.8/10,000 (33 cases). However, during 2012-2020 (late period), the incidence was 7.0/10,000 (68 cases): a 2.5-fold increase. Males were 53.4%. The age range was 1 mo – 17 yrs (median 6 yrs). Of 101 patients, 37 (36.6%) met criteria for definitive and 64 (63.4%) for possible IE. The most common predisposing conditions included congenital heart disease (CHD) (50.5%), central venous catheter (CVC) (25.7%), and immunosuppression (13.9%). CHD was more frequent in the late period (41/68, 60.3%) compared to early period (10/33, 30.3%) (*p* = 0.0059). Cardiac surgery had been performed in 28/51 (55%) prior to IE diagnosis. CVC related infections were more frequent in the early period (16/33, 48.5%) than the late period (10/68, 14.7%), (*p* = 0.0005). Overall, 16 (15.8) cases were culture negative. In culture-positive IE, *S. aureus* was most common (33/101, 32.7%) followed by streptococci (17), *S. epidermidis* (10), Gram negative bacilli (8), Enterococci (7), fungi (5) and HACEK group (4). Causative organisms were similar in both periods except for fungal organisms (5) and *B. henselae* (1) in the late period only. Valve replacement or valvuloplasty were performed in 19 (18.8%) patients. Complications included acute kidney injury (9) and emboli to brain (10) and to lungs (7). Mortality occurred in 15 (14.8%): 8 had CHD, 5 had CVC and 1 had fulminant MRSA infection.

**Conclusion:**

Most of our IE patients had underlying medical conditions. The higher incidence of IE during the late period is likely due to an increase in the number of patients with complex cardiac conditions who underwent surgery at our institution*. S. aureus* was the predominant pathogen followed by streptococci. Mortality rate in our patients was 14.8%.

**Disclosures:**

**All Authors**: No reported disclosures.

